# Evaluation of Osteogenic and Cementogenic Potential of Periodontal Ligament Fibroblast Spheroids Using a Three-Dimensional *In Vitro* Model of Periodontium

**DOI:** 10.1155/2015/605813

**Published:** 2015-11-08

**Authors:** Zurairah Berahim, Keyvan Moharamzadeh, Adrian K. Jowett, Andrew Rawlinson

**Affiliations:** ^1^School of Dental Sciences, University of Science, 16150 Kubang Kerian, Kelantan, Malaysia; ^2^School of Clinical Dentistry, University of Sheffield, Sheffield S10 2TA, UK

## Abstract

The aim of this study was to develop a three-dimensional *in vitro* model of periodontium to investigate the osteogenic and cementogenic differentiation potential of the periodontal ligament fibroblast (PDLF) spheroids within a dentin-membrane complex. PDLFs were cultured in both spheroid forms and monolayers and were seeded onto two biological collagen-based and synthetic membranes. Cell-membrane composites were then transferred onto dentin slices with fibroblasts facing the dentin surface and further cultured for 20 days. The composites were then processed for histology and immunohistochemical analyses for osteocalcin, Runx2, periostin, and cementum attachment protein (CAP). Both membranes seeded with PDLF-derived cells adhered to dentin and fibroblasts were present at the dentin interface and spread within both membranes. All membrane-cell-dentine composites showed positive staining for osteocalcin, Runx2, and periostin. However, CAP was not expressed by any of the tissue composites. It can be concluded that PDLFs exhibited some osteogenic potential when cultured in a 3D matrix in the presence of dentin as shown by the expression of osteocalcin. However the interaction of cells and dentin in this study was unable to stimulate cementum formation. The type of membrane did not have a significant effect upon differentiation, but fibroblast seeded-PGA membrane demonstrated better attachment to dentin than the collagen membrane.

## 1. Introduction

Autologous transplantation of periodontal fibroblasts and stem cells may be a promising technique to induce tissue regeneration in the treatment of periodontal disease [1–4]. Spheroid culture is a form of three-dimensional cell culture that promotes cell-matrix interaction and cellular differentiation without recourse to exogenous growth factors [5] and has been widely used to study cellular differentiation, cell-cell interaction, hypoxia responses, and therapeutically oriented studies [6, 7].

In a recent study, 3D spheroidal cultures of periodontal fibroblasts were developed and characterized* in vitro* with respect to their potential use in conjunction with the biological membranes for periodontal tissue repair and regeneration [8]. It was shown that synthetic and collagen-based membranes were both compatible with periodontal spheroids and stimulated cell proliferation and expression of proteins such as collagen type I, periostin, and Runx2 [8]. However, the interaction of periodontal fibroblast spheroids with dentin has not been thoroughly investigated and it is not clear how the periodontal fibroblasts would behave in a 3D matrix in the presence of both dentin and biological membranes.

Dentin contains a complex mixture of proteoglycans, glycoproteins, sialoprotein, phosphoprotein, and other molecules which are trapped in the mineralized tissue [9]. Experimental studies have accentuated the interactions between dentin, cells from the tooth, and periodontal tissues and reveal dentin to be an adhesive, signaling, and migratory stimulus for various mesenchymal and inflammatory cells. It is believed that dentin exposure, as a consequence of periodontal disease or orthodontic movement, causes release of matrix components which might stimulate the surrounding environment [10].

Therefore, the aim of this study was to develop a three-dimensional* in vitro* model of periodontium including periodontal fibroblast spheroids cultured between dentin and different biological membranes to investigate the osteogenic and cementogenic differentiation potential of the periodontal spheroids within dentin-membrane complex.

## 2. Materials and Methods

### 2.1. Dentin Preparation

Bovine jaws were retrieved from a local abattoir within 4 hours of commercial slaughter from skeletally mature, healthy, normal animals (18 months old). The bovine teeth were extracted using sterile dental forceps and an elevator. Teeth were washed in distilled water and soft tissues were removed with a curette and the pulp was extirpated using a barbed brooch and dental bur under water. The tooth crown was then separated from the root. The enamel was removed using a bone cutter machine under water lubrication (VC-50, LECO) and dentin was sliced to 1 mm thickness. Samples were kept in 0.1% (w/v) thymol (Sigma-Aldrich, Poole, UK) at 4°C until use. Prior to use, the dentin slices were washed 3 times with phosphate buffered saline (PBS) (Sigma-Aldrich, Poole, UK), etched with 30% phosphoric acid for 30 second, washed in PBS, and finally preconditioned for 15 minutes in Dulbecco's Modified Eagle Medium (DMEM) (Sigma-Aldrich, Poole, UK).

### 2.2. Spheroid Culture

Commercially available periodontal ligament fibroblasts (HPDLF, 2630, ScienCell Research Laboratories, CA, USA) were cultured in DMEM supplemented with 10% fetal calf serum, (Sigma-Aldrich, Poole, UK) 2 mM glutamine (Sigma-Aldrich, Poole, UK), 50 U/mL penicillin (Sigma-Aldrich, Poole, UK), and 50 U/mL streptomycin (Sigma-Aldrich, Poole, UK).

Cells from subconfluent culture (passage 6) were released using a solution of 0.05% trypsin (Sigma-Aldrich, Poole, UK) in 0.01% EDTA (Sigma-Aldrich, Poole, UK), counted, and used to generate spheroid culture. Spheroid cultures were initiated by seeding a total of 5 × 10^5^ cells into 96-well plates coated with 1% agarose (Sigma-Aldrich, Poole, UK). Cultures were grown in the same basic media as in monolayer culture with additional L-ascorbic acid (Sigma-Aldrich, Poole, UK) 50 *μ*g/mL. Cultures were incubated in a humidified atmosphere of 5% CO_2_/95% air at 37°C.

### 2.3. Seeding of Spheroids

Spheroids were allowed 24 hours to develop before use. Collagen membrane (Bio-Gide, Geistlich Biomaterials, Switzerland) and polyglycolic acid (PGA) membrane (Synthecon Incorporated, USA) were cut into squares of 10 mm × 10 mm using an aseptic technique. PGA was first disinfected in 100% isopropanol (Sigma-Aldrich, Poole, UK) for 15 minutes followed by 3 rinses in PBS. Membrane squares were placed inside wells of a 12-well plate and preconditioned with fresh complete medium for 10 minutes, twice. Then the medium was discarded and a stainless steel ring (10 mm outer diameter and 6 mm inner diameter) was centrally placed on each membrane. Periodontal spheroids suspended in 200 *μ*L of culture medium were pipetted into each ring and the space surrounding the ring was filled with 1 mL of medium. Spheroids were incubated for 2 days before the ring was gently removed with sterile forceps. Spheroids were allowed to grow on membranes for up to 20 days with a medium change twice a week.

Membranes seeded with monolayer cultures of periodontal fibroblasts were also used as control group.

### 2.4. Dentin-Spheroid-Membrane Culture

The squares of membranes with the attached cells were transferred onto the dentin discs with the fibroblast-cultured side of the membrane facing the dentin. The dentin-cells-membrane complexes were cultured for another 20 days in the complete culture medium.

### 2.5. Histological Processing

At the end of culture, the dentin-cells-membrane complexes were washed three times for 10 minutes using PBS and fixed in 10% buffered formalin (Genta Medical, York, UK). Then, they were decalcified in 14% aqueous EDTA for 14 days. The decalcified samples were dehydrated in serial dilution of ethanol before being embedded in paraffin wax. Samples were sectioned to 5 *μ*m thickness using a microtome (RM2235, Leica, Germany) and dried in a hot air oven for 1 hour at 60°C. Then they were rehydrated and stained with hematoxylin and eosin (H&E).

### 2.6. Immunohistochemistry

Protein detection was carried out using immunostaining against osteocalcin, Runx2, periostin, and cementum attachment protein (CAP).

The sections were pretreated with 10 mg/mL hyaluronidase (Sigma-Aldrich, Poole, UK) in PBS for 30 minutes at 37°C, followed by 2 mg/mL pronase (Fluka Biochemika, UK) in PBS for 30 minutes at the same temperature. In the case of Runx2, trypsin was used as an additional antigen retrieval stage at 37°C for 20 minutes. Sections were washed with PBS followed by quenching endogenous peroxidase activity with 3% v/v in 50% methanol (Fisher Scientific, Loughborough, UK) followed by washing with tris buffered saline (TBS) (Sigma-Aldrich, Poole, UK). Sections were then exposed to 3% w/v bovine serum albumin (BSA) (Sigma-Aldrich, Poole, UK) in TBS/tween20 for 1 hour to avoid nonspecific staining. Samples and positive controls were incubated with primary antibody overnight at 4°C including mouse monoclonal osteocalcin 10 *μ*g/mL (ABCAM, MA, USA), Rabbit polyclonal periostin 1 *μ*g/mL (ABCAM, MA, USA), rabbit polyclonal Runx2 2 *μ*g/mL (ABCAM, MA, USA), and mouse monoclonal CAP 1 *μ*g/mL (Santa Cruz Biotechnology, CA, USA).

For the negative control, sections were incubated with 1% BSA in TBS/tween20.

All sections were washed with TBS and incubated with the appropriate secondary antibody for 1 hour at room temperature. The appropriate secondary antibody was prepared using a biotinylated secondary antibody kit (VECTASTAIN ELITE ABC, Vector Laboratories, Peterborough, UK) and the subsequent technique according to the manufacturer's instructions. Sections were then exposed to peroxidase substrate solution (DAB-SK-4 100, Vector Laboratories, Peterborough, UK) for the visualization of protein. Counterstaining was carried out in Harris's hematoxylin.

## 3. Results

### 3.1. General Appearance

**Figure 1:**
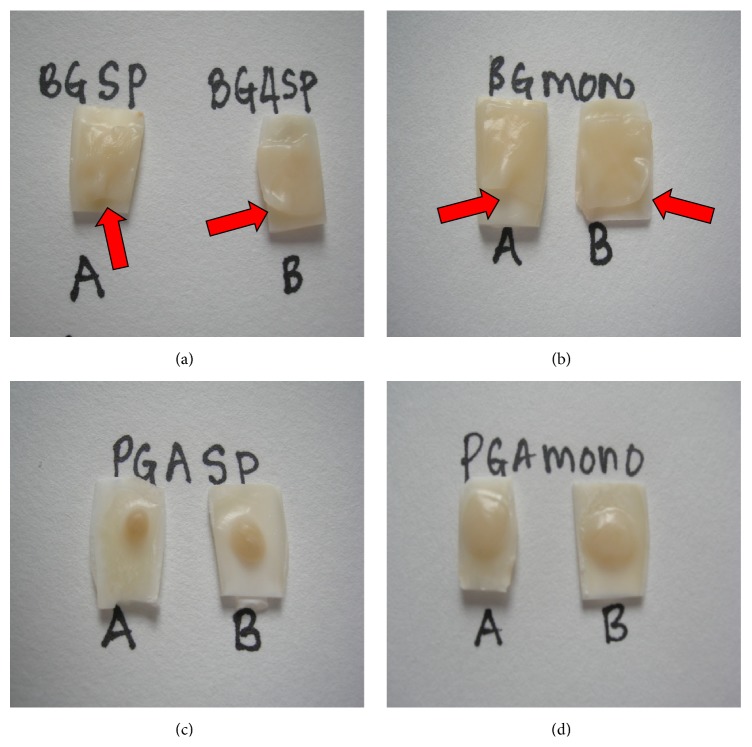
The appearance of PGA and collagen on dentin after 20 days in culture. (a) Spheroid-collagen, (b) monolayer seeded collagen, and (c) spheroid-PGA (d) monolayer seeded-PDLF PGA. Red arrows show the incomplete attachment of collagen on dentin.

Generally both membranes adhered well on dentin surface. At 20 days, PGA cultured with either periodontal spheroids or monolayers showed a better attachment on dentin compared to collagen membrane. PGA showed a well-blended appearance and complete attachment onto the dentin surface; however collagen membrane showed some nonadherent areas to the dentin surface mainly on the margins of the membrane (Figure 1).

### 3.2. Histological Appearance

#### 3.2.1. Collagen-Spheroid-Dentin Complex

**Figure 2:**
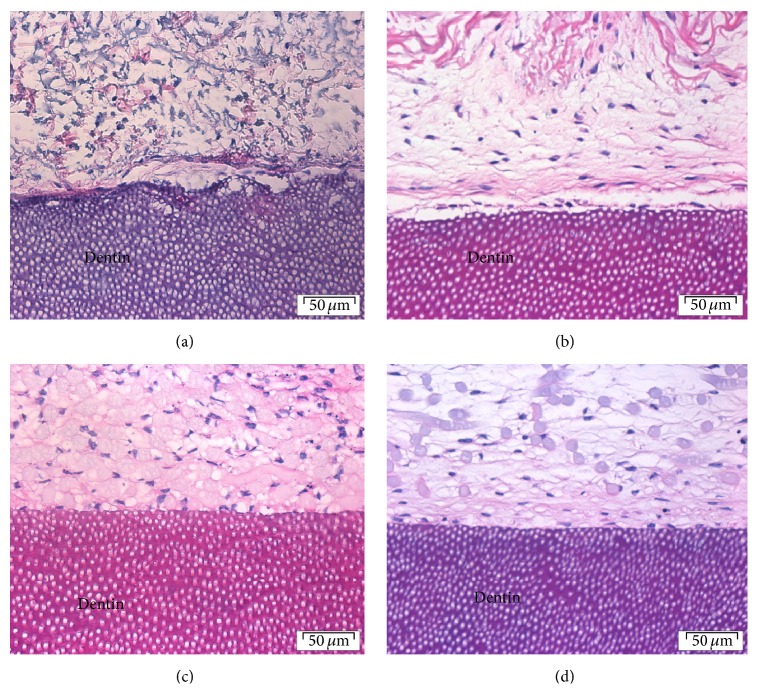
Histological sections of (a) collagen-spheroids-dentin, (b) collagen monolayer fibroblast-dentin, (c) PGA-spheroid-dentin, and (d) PGA-monolayer fibroblast-dentin cultures after 20 days. Hematoxylin and eosin, original magnification ×40 (scale bar = 50 *μ*m).

Histological examination of the collagen membranes seeded with either spheroids or monolayer fibroblasts showed presence of fibroblasts spread across the collagen membrane and at the interface between the membrane and the dentin surface (Figures 2(a) and 2(b)). In a small proportion of sections there was evidence of separation of the membrane from the dentin surface in some areas.

#### 3.2.2. PGA-Spheroid-Dentin Complex

H&E sections of both spheroids and monolayer cells on PGA membrane cultured on dentin showed closer attachment to the dentin surface compared to that of collagen membrane.

Proliferation and ECM production were evident and flattened cells were seen lining the surface. The fibers of the PGA membrane were clearly visible with presence of cells and matrix (Figures 2(c) and 2(d)).

### 3.3. Protein Expression

#### 3.3.1. Osteocalcin Detection in Collagen-Spheroid-Dentin Complex

**Figure 3:**
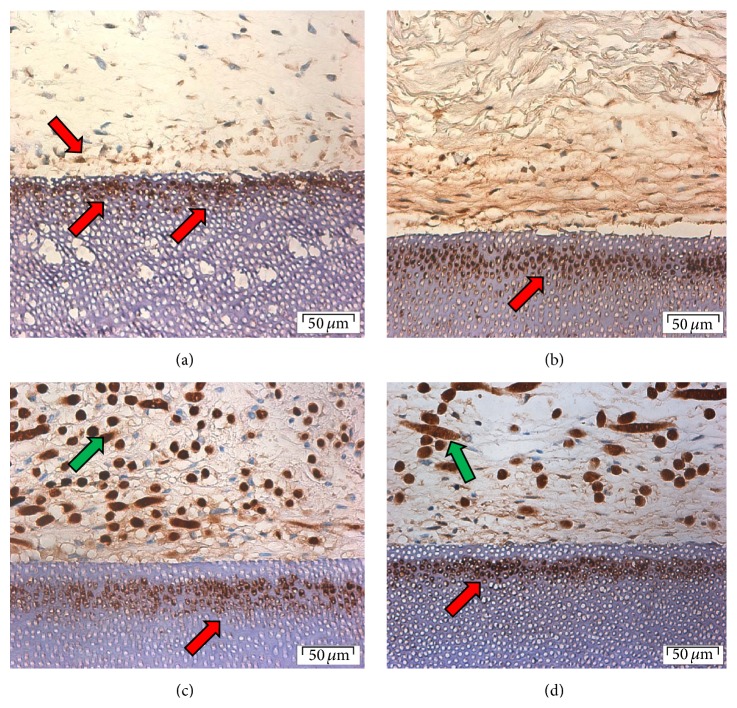
Osteocalcin expression in (a) collagen-spheroids-dentin, (b) collagen monolayer fibroblast-dentin, (c) PGA-spheroid-dentin, and (d) PGA-monolayer fibroblast-dentin cultures after 20 days. Original magnification ×40 (scale bar = 50 *μ*m). Green arrows show nonspecific staining.

Osteocalcin was detected on the surface of collagen membrane seeded with PDLF spheroids. No obvious staining was seen in the bulk of the membrane. A low level of staining was detected in the matrix at the surface of cells-membrane facing the dentin and more intense staining was detected inside the dentinal tubules and matrix slightly deep to the junction between membrane and dentin (Figure 3). PDLF monolayer seeded onto collagen membrane and cultured on dentin showed a similar pattern of osteocalcin expression to that seen with spheroids. However at the interface between membrane and dentin the staining was more intense and through a greater depth of the cell-membrane construct. A layer of cellular fibrous matrix showed a gradient of staining, increasing towards the dentin. There was also marked staining of osteocalcin within dental tubules in a band of around 30 *μ*m, deep to the adjacent membrane. Notably, the dentin adjacent to the cellular layer was not stained for osteocalcin.

#### 3.3.2. Osteocalcin Detection in PGA-Spheroid-Dentin Complex

Osteocalcin primary antibody bound nonspecifically to the PGA fibers. Expression of osteocalcin by cells from spheroids within a PGA membrane was broadly similar to that seen previously (Figure 3(c)). Staining of osteocalcin within the dentin was especially prominent. A low level of staining was found in the matrix; however this was absent from superficial 75 *μ*m of the dentin-cell-membrane contact. PDLF monolayer seeded PGA membrane expressed osteocalcin where it was stained within the extracellular matrix. Intense staining was present in the dentin deep to the membrane but not in the dentin adjacent to the seeded membrane (Figure 3(d)).

#### 3.3.3. Runx2 Detection on Spheroid-Collagen-Dentin Complex

**Figure 4:**
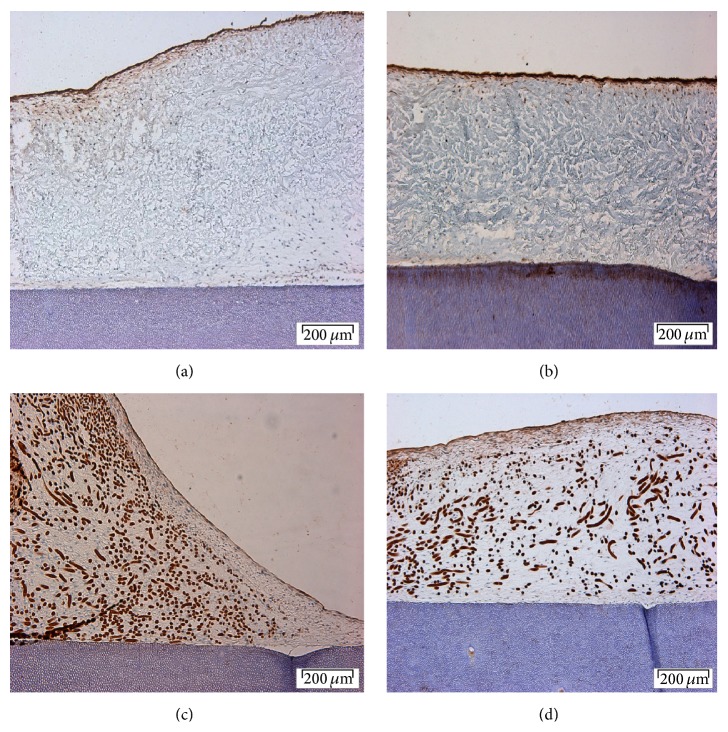
Runx2 expression in (a) collagen-spheroids-dentin, (b) collagen monolayer fibroblast-dentin, (c) PGA-spheroid-dentin, and (d) PGA-monolayer fibroblast-dentin complex cultures after 20 days. Original magnification ×10 (scale bar = 200 *μ*m).

Staining was detected in the superficial cells of spheroids in collagen membrane. There was no expression either in the centre of the membrane or at the dentin interface (Figure 4(a)).

PDLF monolayer cells seeded into collagen membrane also showed marked staining in the superficial layers. A band of staining was also seen within dentin at the interface (Figure 4(b)).

#### 3.3.4. Runx2 Detection on Spheroid-PGA-Dentin Complex

Cells from spheroids in PGA showed a very low level of expression in the superficial layers only (Figure 4(c)). PDLF monolayer cells seeded into PGA on dentin expressed Runx2 in a wider superficial band than seen for collagen membrane (Figure 4(d)).

#### 3.3.5. Periostin

**Figure 5:**
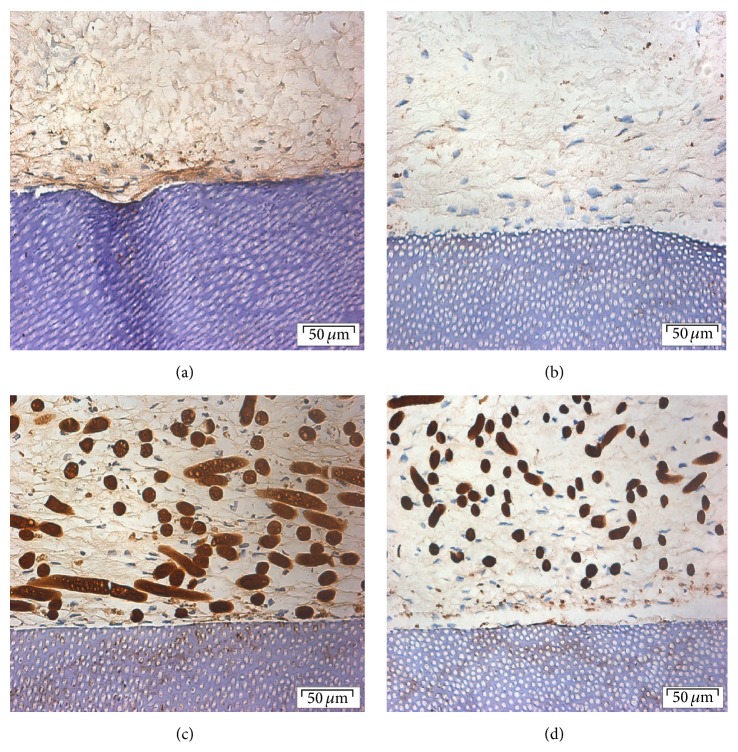
Periostin expression in (a) collagen-spheroids-dentin, (b) collagen monolayer fibroblast-dentin, (c) PGA-spheroid-dentin, and (d) PGA-monolayer fibroblast-dentin cultures after 20 days. Original magnification ×40 (scale bar = 50 *μ*m).

Periostin expression was seen in the matrix throughout the construct in all four culture combinations (Figure 5). In each case the level of expression was greatest around superficial cells. It was possible to detect a greater level expression in the matrix adjacent to the dentin in the case of spheroid and in some regions of the cell-PGA-dentin construct. Occasionally a layer of dentin staining was seen but no pattern could be discerned.

#### 3.3.6. Cementum Attachment Protein

**Figure 6:**
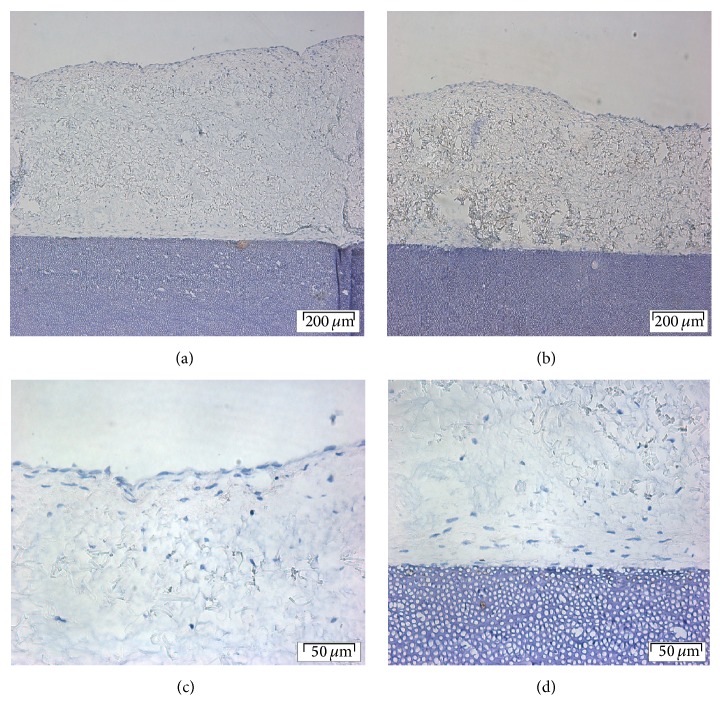
CAP expression in spheroids-collagen-dentin complex: (a) an overview of collagen and dentin, (b) negative control, (c) a closer view of the surface of collagen, and (d) a closer view of collagen and dentin junction. Scale bar of (a) and (b) = 200 *μ*m, magnification: ×10; (c) and (d) = 50 *μ*m, magnification ×40.

Expression of cementum attachment protein was not detected in either PGA or collagen membrane with cells from either monolayer or spheroids culture.  Figure 6 shows that no staining above background was present for spheroid-derived cells on collagen. Figures from the other 3 experiments condition are not shown.

## 4. Discussion

The results of this study have shown that membranes seeded with PDLF-derived cells adhered to dentin but the dentin had a limited effect on differentiation of the cells. No clear differences between cells from monolayer or spheroids were seen nor were there significant differences in behavior between collagen and PGA membranes. However it has been shown that both spheroids and monolayer cells-PGA membrane attached better to the dentin than collagen membrane. In both cases the attachment is likely to be solely an adhesion between connective tissue and dentin rather than a true periodontal regeneration as neither insertion of fibers into dentin nor cementum secretion was seen by light microscopy. This finding is similar to that of an* in vivo* study using connective tissue grafts on the root surfaces [11]. In their study, a connective tissue graft was placed on root dentin* in vivo* for 6 months. Although there were some areas with true regeneration, the authors reported that most other areas presented with only connective tissue adhesion. There is also a report* in vitro* which showed an increase in proliferative activity and alkaline phosphatase activity when primary osteoblast cells were cultured on natural calcified dentin [12]. These findings showed that calcified dentin serves as an excellent substrate for cell adhesion.

Osteocalcin is a well-established marker of bone, dentin [13], and cementum synthesis [14] and thus its expression within a PDLF construct is expected. The cell-membrane constructs were opposed onto dentin with the intention of stimulating differentiation along a cementoblast-like lineage. In this regard, a slight increase in staining intensity of the basal cell layers might suggest that this has occurred. More strikingly, osteocalcin staining was strong in the dentin deep to the cells. As dentin peripheral to the cell-membrane was not stained, and the staining was parallel with the cell-membrane, it can be postulated that osteocalcin is being secreted by the cells. As the staining is in a layer at least 20 *μ*m deep to the cells, it is most likely that secreted osteocalcin has diffused into the dentin slice and bonded to the deeper surface within the slice. As the dentin was pretreated with 30% phosphoric acid there would be a surface of exposed dentinal tubules. The use of etchant was based on the concept that biochemical modification of the root surface could enhance cell migration and attachment to an exposed root surface [15, 16]. It has also been reported that exposed dentin together with bone morphogenetic protein (BMP) is capable of inducing cementogenesis* in vivo* [17]. However despite these findings, it is debatable whether demineralization has a significant effect on reattachment clinically.

The increased expression of Runx2 distant to the dentin interface suggests that it is unlikely that dentin is exerting an inductive influence. Runx2 can be induced by members of the TGF-*β* family [18], which are likely to be expressed within a population of differentiating mesenchymal cells. In turn, Runx2 can induce dentinogenesis and osteogenesis [19]. The findings of this study are consistent with the observations of Ducy et al. [20] who demonstrated that expression of Runx2/cbfa1 in mouse embryo was not detectable before mineralization occurred. In this experiment there was no evidence of mineralization taking place which could be due to the lack of other mineralization factors. Therefore, it is also doubtful whether the Runx2 detection on the surface of cell-dentin-construct is really specific. There is a possibility of nonspecific staining of Runx2 which is explained by the atypical staining pattern which is not seen in spheroid or other areas of the cell-dentin construct.

*β*ig-h3 which belongs to the same group as periostin has been demonstrated to inhibit mineralization of the periodontal ligament [21]. Ohno et al. showed that in the presence of vitamin D3, the level of *β*ig-h3 mRNA was markedly decreased. They also demonstrated that recombinant *β*ig-h3 suppressed the alkaline phosphatase activity and bone nodule formation of cultured PDL cells. The absence of mineralization in these experiments may be partly accounted for by the expression of *β*ig-h3 like protein indicated by generalized staining with an anti-periostin antibody.

The absence of cementum attachment protein in this study indicates that neither calcified dentin nor a layer of exposed dentin alone was able to stimulate the periodontal fibroblasts to differentiate along the cementogenic lineage, although they arise from the same origin [22]. This is supported by Thomas and Kollar [23] who demonstrated that the combination of exposed dentin surface and follicular cells is not a sufficient stimulus for cementoblast differentiation and cementogenesis.

Since bovine dentin used in this study has different morphological, chemical composition, and physical properties compared to human dentin [24], further studies using human dentin would be recommended to confirm the findings of this study.

In this study spheroids were allowed to grow on membranes for up to 20 days prior to seeding on dentin slices. This could potentially initiate differentiation of the spheroids prior to dentin seeding. Longitudinal studies are required to assess the effect of different seeding times on differentiation potential of the spheroids.

3D* in vitro* model of periodontium reported in this study contained dentin, PDL fibroblasts, and soft-tissue membranes. However, this model lacked bone and epithelial components of normal periodontium. Further research is underway to optimize the existing* in vitro* periodontal model by incorporating tissue engineered bone and oral mucosa to the 3D system. The advanced tissue engineered model can be used as a clinically relevant test system for the investigation of periodontal disease and the effects of different treatment modalities.

## 5. Conclusions

Periodontal ligament fibroblast cells exhibit some osteogenic potential when cultured in a 3D environment in the presence of dentin as shown by the expression of osteocalcin. However the interaction of cells and dentin in this study was unable to stimulate cementum formation. PGA and collagen membranes did not have a significant effect upon differentiation, but fibroblast seeded-PGA membrane demonstrated better attachment to dentin in comparison to fibroblast seeded-collagen membrane.
